# Implementing Personalized Cancer Medicine: Insights from a Qualitative Interview Study

**DOI:** 10.3390/jpm15040150

**Published:** 2025-04-09

**Authors:** Michele Masucci, Jenny Del Villar Pérez, Pamela Mazzocato, Ingemar Ernberg, Mats Brommels

**Affiliations:** 1 Department of Microbiology Tumor and Cell Biology (MTC), Karolinska Institutet, Biomedicum Q8C, Solnavägen 9, 171 65 Solna, Sweden; ingemar.ernberg@ki.se; 2 Department of Learning, Informatics, Management and Ethics (LIME), Medical Management Centre, Karolinska Institutet, Tomtebodavägen 18a, 171 65 Solna, Sweden; jenny.perez@live.se (J.D.V.P.); pamela.mazzocato@ki.se (P.M.); mats.brommels@ki.se (M.B.)

**Keywords:** personalized medicine, precision medicine, personalized cancer medicine, cancer research, cancer healthcare, precision oncology, implementation research, translation research, healthcare organization, medical management

## Abstract

**Background**: Personalized cancer medicine (PCM) tailors cancer treatments based on individual genetic profiles, enabling more precise and effective therapies. Despite its potential, integrating PCM into clinical practice remains challenging because of organizational and systemic barriers. This study examined the factors influencing PCM implementation at a major cancer center in Stockholm, Sweden. **Methods**: We conducted semi-structured interviews with 16 medical professionals and management staff from Karolinska University Hospital and Karolinska Institutet. Content analysis was used to identify key themes related to PCM implementation. This study followed the established Consolidated Criteria for Reporting Qualitative Research guidelines to ensure methodological rigor and transparency. **Results:** Informants framed PCM as both a technological innovation and a patient-centered approach. However, significant barriers to implementation were identified, including organizational inertia, fragmented funding models, and ethical challenges related to access and equity. Structural silos between academic and healthcare institutions complicate integration. Key facilitators include leadership commitment, cross-sectoral collaboration, and a supportive policy environment. Participants emphasized the need for integrated infrastructure, real-time data-sharing mechanisms, and interdisciplinary training programs to support PCM. **Conclusions**: Successful PCM implementation requires overcoming entrenched organizational and systemic barriers through a multi-stakeholder approach involving healthcare providers, researchers, policymakers, and patient advocates. The findings underscore the necessity of a “third-form organization” to mediate between academia and clinical care. Addressing these challenges requires adaptive governance models, evidence-based policy reforms, and sustainable funding frameworks. Future research should explore comparative contexts to enhance the scalability and generalizability of PCM integration strategies.

## 1. Introduction

Advances in molecular cell and tissue biology and the refinement of sequencing technology have ushered in an era of precision medicine where therapeutic strategies are tailored to each patient’s genetic makeup [1]. In oncology, personalized cancer medicine (PCM) promises improved treatment outcomes by designing therapies that target the unique molecular abnormalities of individual cancers [2]. While the potential of PCM is clear, its implementation in clinical settings presents multiple challenges, including technological readiness, workforce training, financial constraints, ethical dilemmas, and building patient-centered treatment teams [3,4,5]. Proper assessment of outcomes is limited by the disparate understanding of the concept and its implications among different actors [6,7,8].

A PubMed search using the keywords “personalized”, ”cancer”, and “medicine” yields 40,000 hits with more than 7000 articles published between 2023 and 2025. Through this vast literature, multiple challenges to the successful integration of PCM into clinical practice have been identified, as outlined in a recent review [9]. The financial implications of personalized therapies, which are often associated with high costs, have been highlighted [10]. Ethical considerations, especially concerning genetic data privacy and the management of incidental findings, have been explored [11,12,13,14]. The need for interdisciplinary collaboration has been emphasized, along with the importance of integrating patient perspectives into precision medicine paradigms [15,16,17,18]. The effective management and sharing of resources, as well as adequate support for clinical investigators, are essential for driving PCM forward [19,20,21]. These studies often provide some insight into the impact of specific institutional contexts on the translation process, but a precise understanding of how institutional settings influence PCM implementation is still lacking. Many studies focus on national trends or generic barriers, providing limited detail on how implementation is shaped by specific institutional settings, governance structures, or healthcare systems [1]. While recent qualitative research has begun to explore stakeholder perspectives, it often focuses on single roles (e.g., clinicians) or lacks regional specificity [22,23]. Consequently, little is known about how local policies, institutional interplay, and healthcare infrastructure jointly influence PCM uptake—highlighting the need for detailed, context-sensitive investigations.

Studies and policy analyses have identified persistent, practical challenges to PCM integration—including inadequate data infrastructure, fragmented diagnostic pathways, misalignment with existing policies, divergent stakeholder interpretations of evidence, limited patient engagement, and concerns about equity in access [24,25,26,27,28]. However, the qualitative evidence base remains fragmented and insufficient to guide effective implementation. In particular, there is a need for systematic, context-specific inquiry into the perspectives of key stakeholders—clinicians, patients, NGOs, and health system decision-makers—who are all critical to PCM adoption [29,30].

This study explored how participants perceived and navigated the challenges and opportunities that arose during the PCM implementation process. The dynamics of the Stockholm region, encompassing all relevant stakeholders, including Karolinska Institutet (KI), a prominent biomedical education and research center; the Karolinska University Hospital (KUS), a leading tertiary care unit of the Region Stockholm (RS); policymakers; patient advocates; and pharmaceutical industry representatives, provided a distinct backdrop for this analysis.

## 2. Materials and Methods

### 2.1. Study Setting

This study was performed within the local framework of Region Stockholm (RS), a public authority governed by elected politicians responsible for providing healthcare and supporting clinical research. The Karolinska University Hospital (KUH), a unit within the RS healthcare organization, is a major university hospital with over 16,000 employees and 1–4 million patient visits annually. Many medical staff are engaged in clinical research. Karolinska Institutet (KI) is one of the largest medical universities in Europe, with approximately 6500 full-time students and approximately 350 doctoral degrees granted annually. The use of RS’s care-providing units for professional education and clinical research performed by KI is regulated by a mandatory agreement between KI and RS wherein clinical teachers have joint appointments at KI and KUH. In 2019, the chief executive officer of KUH and the president of KI jointly decided that Precision Medicine (PM) should be the overriding theme of collaboration in clinical research and academic healthcare (https://news.ki.se/ki-and-karolinska-university-hospital-establish-joint-precision-medicine-center, accessed on 12 March 2025).

### 2.2. Participants and Recruitment

A purposeful sample of 20 medical professionals and managers directly involved in the implementation of PCM was identified based on their specialized knowledge and diverse institutional roles. Sixteen individuals consented to participate in the study. These represented a range of perspectives essential to understanding the organizational and translational processes involved in PCM adoption. This sampling strategy aligns with the principles of information power [31], prioritizing the relevance and richness of each participant’s contribution over numeric representativeness. Participation was voluntary, with written informed consent obtained and all personal identifiers removed to ensure anonymity, in accordance with the process approved by the Swedish Ethical Review Authority (Dnr 2020-01750). The strategic sample included senior physicians, translational researchers, clinical oncologists, academic leaders, and hospital managers, all with mandates to advance PCM within their institutions and roles. This focused yet professionally diverse sample was designed to maximize data richness and capture nuanced, practice-based insights from those with profound and practical engagement in PCM. To mitigate selection biases, we identified persons with clear roles and mandates to work towards PCM implementation at their institution. Moreover, we ensured a broad representation of professional roles to the extent possible. The composition of the participants is listed in Table 1.

The final sample size of 16 participants was sufficient to support the aims of this study, in line with methodological guidance suggesting saturation occurs when the sample is information-rich and thematically focused [32,33]. The relatively homogeneous professional context of the participants, i.e., their shared mandate and active involvement in PCM implementation, contributed to reaching data saturation.

### 2.3. Data Collection and Analysis

Through in-depth interviews with key stakeholders, we aimed to gain insight into the readiness, challenges, ethics, and impact of the local ecosystem on the assimilation of PCM into cancer care. This qualitative study was based on semi-structured interviews that were subjected to conventional content analysis [34]. The Consolidated Criteria for Reporting Qualitative Research (COREQ) guidelines [35] were followed (see Supplementary File S1). The first author conducted the interviews between January 2020 and June 2024, using an interview guide (see Supplementary File S2) featuring the factors influencing the PCM translation process. The domains explored in the interviews are listed in Table 2.

In-person or telephone interviews began with predefined questions, followed by questions aimed at capturing unique insights from interviewees. The interviews lasted 40–60 min and were recorded digitally. The recordings were transcribed verbatim using the MyGoodTapes^®^ software. Transcriptions were checked by the first and second authors against the recordings and corrected when necessary. Data collection stopped when no new concepts, themes, or insights relevant to the study’s aims emerged in the final interviews, which was when data saturation was reached. Saturation was assessed through ongoing comparative analysis, team triangulation, and iterative coding, consistent with established qualitative methodologies [32,36]. The transcripts were scrutinized through conventional content analysis using an inductive approach [34]. The first and second authors repeatedly read the transcripts to reach immersion. To ensure methodological rigor and reflexivity, the first, second, and last authors independently analyzed two randomly selected transcripts to establish analytic consistency and validate emergent patterns. This investigator triangulation mitigated potential selection bias and served as a checkpoint for verifying that data saturation had occurred. The absence of new codes or meaning units in the final interviews reinforced the conclusion that thematic comprehensiveness had been achieved. Subsequently, the second author systematically analyzed all transcripts, transferred marked meaning units to a Microsoft Excel© document together with their extracted whole paragraphs, and assigned them a semantic code (label). The codes were posted on a MIRO^®^ dashboard and discussed by all authors for categorization and re-categorization until a consensus was reached. These categories were named and grouped into themes. Citations to illustrate categories were collected using an Excel spreadsheet. Member checking was conducted by circulating emerging themes, categories, and draft manuscript with all study informants, all of whom confirmed the resonance and completeness of the analysis. Their responses were documented and no additional themes were suggested, further corroborating that data saturation was not only reached but that the final thematic structure was credible and representative.

## 3. Results

The results are presented as themes, with descriptions based on the categories and subcategories identified. Illustrative quotes from the interviews are provided. The themes and categories that emerged from the thematic analysis are presented in Table 3. Full thematic analysis, including sub-categories, can be accessed through Supplementary File S3.

### 3.1. Theme 1—Different Conceptions of PCM

Analysis of the interviews revealed that the concepts of “personalization” and “precision” in cancer care were understood differently across communities. This influences the application of concepts in the development of new cancer treatments. While some professionals used these terms interchangeably, some identified “precision medicine” with molecular profiling, whereas according to others, “personalized medicine” encompasses a broader range of approaches, including patient symptoms.

“*There are many who argue that precision medicine is more narrowly focused, while personalized medicine is broader.*”

(Hospital Management, Code 14)

“*Technologically, it is definitely the Next Generation Sequencing-based technology that has kick-started the entire field of precision medicine.*”

(Academic Leadership, Code 16)

### 3.2. Theme 2—Complex and Dynamic Relationships Between Actors

The development of PCM involves a complex network of healthcare providers, researchers, developers, regulators, funders, and care receivers. It is well represented by the Penta Helix model [37], which is a collaborative framework involving the government, academia, industry, civil society, and the media working together to drive innovation, sustainable development, and societal progress. A majority informants stressed that, while essential for driving PCM, effective collaboration among key parties faces fundamental challenges. Most importantly, the two main legal entities, healthcare and academia, operate under different employment conditions and have different priorities, objectives, and internal dynamics. This poses challenges for translational projects. Reimbursement systems are primarily designed for routine healthcare, not research and development, creating gaps between innovation and practice. Some informants also stressed that while the engagement of the pharmaceutical and biotech industries is essential, in-house competence must be preserved and expanded.

“*I wish there was more courage to try having a consortium between industry, academia, and all the five sectors in the Penta Helix. They need to be at the table.*”

(Academic Leadership, Code 16)

“*We need a common organization, not necessarily a legal entity, and a clear division of roles.*”

(Senior Researcher, Code 10)

“*Then there are the passionate individuals who make this work.*”

(Senior Researcher, Code 15)

The informants, especially senior physicians and researchers, emphasized the need for strong, visionary leadership capable of uniting different stakeholders towards common goals, fostering collaboration, and driving organizational change. The leadership of all the involved institutions’ concerted support for collaboration fostered synergies. When the conditions for cooperation are established, change becomes a motivating factor, and single individuals are the driving forces that bridge structural divides and find new ways forward.

“*Collaboration is the key word here, a good collaboration between academia and healthcare. We can’t do this alone; we have to do it together.*”

(Senior Physician, Code 3)

The implementation of PCM requires specific actions. Several informants pointed to the establishment of joint KI-KUH units, such as the Precision Medicine Centre Karolinska (PMCK) and the Personalized Cancer Medicine Program, which have a clear mandate to facilitate the process. A theme strongly emphasized by some informants was how despite challenges, the drive of individual stakeholders, combined with effective leadership, can overcome obstacles and ensure the successful integration of PCM into modern healthcare.

“*This is a transformation; you have to change the organizational model and the operational model. The entire leadership line must pull in the same direction.*”

(Academic Leadership, Code 6)

“*It’s collaboration, more competencies. It requires a different way of thinking about how healthcare should be organized and funded. And especially within PCM and cancer. Because there are a lot of expensive drugs, both in diagnostics and treatment.*”

(Senior Physician, Code 5)

### 3.3. Theme 3—Appropriate Technologies and Structures

A recurring statement among the informants was that PCM hinges on the development and coordination of emerging technologies, expertise, and shared infrastructure. While the research infrastructure is largely in place, technologies are rapidly advancing, which requires the development of specific competencies and capabilities, particularly in the areas of multiomics and bioinformatics. Senior researchers have pointed out the critical need for coordinated efforts in data management, computing, and resource sharing. Organizational arrangements, including the expansion of clinical trial capabilities, are necessary to bridge the gap between routine health care and translational research.

“*We worked for ten years before we got the diagnostics really up and running, and it’s based on the exceptional expertise of many people across several different disciplines.*”

(Senior Physician, Code 7)

“*But there is also a significant resource, such as research nurses, who are key to this. Therefore, they must be trained in precision medicine.*”

(Hospital Management, Code 9)

Emerging technologies are rapidly advancing, which will significantly impact the PCM landscape. There is a critical need for coordinated efforts in methodology, conceptual development, and the creation of shared infrastructures for data management, computing, and resource pooling among stakeholders in order to make use of advancements to the benefit of patients.

“*There is a need to coordinate methodologically and conceptually, develop shared infrastructures for data management, computing, and share/pool resources across stakeholders.*”

(Hospital Management, Code 12)

A majority of informants stressed that the successful implementation of PCM requires dedicated training for key professionals such as research nurses, doctors, and pathologists. New professionals, such as molecular pathologists and bioinformaticians, should be established to meet growing demands and provide opportunities for capable individuals to contribute to PCM. Beyond the essential clinical functions, dedicated translational researchers, IT and data specialists, management, and legal and health economy expertise play crucial roles in fostering implementation.

“*We also need to implement and provide training for doctors, pathologists, and everyone else involved in this.*”

(Senior Researcher, Code 11)

### 3.4. Theme 4—New Organizational Forms Facilitate Implementation

Most senior physicians and researchers have emphasized the need for new organizational models and collaborative frameworks, enabling a continuum from bench to bedside. This includes fostering multidisciplinary cooperation and providing further education. The organization must extend across sectors, including academia, industry, healthcare, and the government.

“*One must find organizational solutions, as multiple specialists need to collaborate, including staff doing diagnostics, clinicians, and informaticians so that a working method can be established.*”

(Senior Physician, Code 3)

Transdisciplinary collaboration is essential and entails increasing the number of clinician researchers with dual KUH-KI affiliations and establishing new professional roles. New working methods are needed to better integrate research into the hospital environment. KUH must adopt incentive structures that support the translational research process more effectively.

“*Have some sort of office that can work on overarching issues, such as legal matters related to the data department, the data department itself, and certain central issues relevant to all initiatives that require national coordination.*”

(Academic Leadership, Code16)

Some informants shared the need for organizations that can facilitate relations between stakeholders. The modernization of healthcare towards PM requires new forms of coordination of national infrastructure that unites regional and national efforts; a dedicated office should address overarching issues. According to several informants, organizational culture must promote scientific innovation, provide legal and data support, and be free from immediate production pressure.

“*We believe that either an infrastructure or a national program is needed to serve as an umbrella organization for PCM, where regional and national entities can come together, along with some sort of office that can work on overarching issues.*”

(Research Leadership, Code 16)

### 3.5. Theme 5—Political Engagement and Legislative Efforts

The organization and governance of KUH and KI are perceived to be suboptimal when implementing PCM. Political decision-makers prioritize the delivery of advanced healthcare at KUH, while academic leadership focuses primarily on research and education. This fragmented and rigid organization hinders translational research. Some informants also mentioned how outdated legal frameworks prevent maximizing the benefits of healthcare and research investment. Addressing these issues requires political engagement and reforms guided by medical expertise and is informed by international examples.

“*In the other world [healthcare], everything is very politically driven and short-term focused. The entire healthcare system is governed by the same principles. It’s a large production apparatus.*”

(Senior Physician, Code 01)

“*Healthcare is very slow to change, with rigid systems in place for reporting, financing, and management. It’s not easy to simply start working in entirely new ways.*”

(Operations Developer, Code 5)

The need to fulfill legal requirements while maintaining research progress and innovation is perceived as a key challenge. Frameworks like the General Data Protection Regulation (GDPR) and In Vitro Diagnostics Regulation (IVDR) protect individual rights, but the lack of legal counseling supporting complex interpretation and adherence adds administrative burdens. The current Swedish legislation on the secondary use of healthcare data is ill-suited for big data analysis, and unclear guidelines hinder compliance. While the laws are well-intentioned, the cost of compliance is often underestimated. To address these challenges, legal expertise and infrastructure must be strengthened.

“*For instance, the legal framework is highly protective, which is generally good, but at times it can become a barrier.*”

(Senior Researcher, Code 6)

Since political interests and commitments shape the conditions for translational research and its integration into healthcare, the advancement of PCM rests on a stronger political will and a long-term focus. Some informants expressed concerns that Sweden is falling behind in genomics-based medicine, making international collaboration essential for enhancing local PCM capabilities.

One informant pointed out that due to Sweden’s small size and fragmented organizational structure, engagement must be continually justified to global funders and investors. This poses a challenge to attract, initiate, and maintain innovation.

“*The problem is that the global level often comes into play quickly within each company. And this means that you must defend why the global level should invest in Sweden.*”

(Senior Physician, Code 7)

### 3.6. Theme 6—Financial Preconditions for Translational Research

Throughout the interviews, differences in funding and production models were identified as important hindrances to the implementation of PCM. Regional administration supports university healthcare, whereas academic research and education are supported primarily by state and private research funds. The gaps produced by the different modes of resource allocation should be addressed. Informants have identified the need to combine various funding sources to overcome organizational differences and promote translational processes. Clinicians interested in translational researchers and PCM have sometimes managed to fill this gap; however, to achieve sustainability, all stakeholders need to revise their organizational structures to improve their financial conditions.

“*One would like the incentive structure in academia to become more, perhaps, implementation-oriented. What does it mean for the patient? It’s difficult to find those markers that are good.*”

(Operations Developer, Code 5)

“*If I take the X study as an example, it’s a study conducted in four countries that has raised about 100 million SEK from various stakeholders, primarily companies that sponsor medication; without that, it wouldn’t be possible. We allocate some of it to treatment research. The research grants are too small compared to what it costs.*”

(Senior Researcher, Code 9)

The requirement for significant infrastructure investment and the high cost of PCM treatment are the major challenges. Although academic research is crucial for advancing PCM, the structural barriers between academia and healthcare hinder the effective use of resources. Current funding models are often short-term and unsuitable for translational clinical trials, whereas the focus of healthcare on production often neglects the potential benefits of academic research. Health economics studies are required to ensure that PCM investments are cost-effective and provide a clear return on investment.

“*One must look at precision medicine in cancer; then you can surely present and conduct a socio-economic analysis, and it seems that not much has been done on that.*”

(Operations Developer, Code 5)

“*That is the challenge. The cost will arise somewhere, but the savings will appear in a third or fourth place in another budget.*”

(Senior Physician, Code 12)

### 3.7. Theme 7—Patients’ Participation, Ethics, and Equity

All interviewees stressed that patient participation was central to PCM success. The data-intensive PCM approach requires patient participation for data sharing. In addition, patient-reported data and their active participation in decision-making are crucial in ensuring that the patient’s perspectives and experiences shape the data collection process and the overall care they receive.

“*Extremely important that patient organizations actually gain influence.*”

(Hospital Management, Code 9)

“*To achieve precision cancer medicine, we likely need to inform the public that we will need to share more and more data, and whose role is it to do this?*”

(Senior Physician, Code 11)

A couple of informants highlighted national disparities in access to advanced cancer care, emphasizing the importance of centralizing resources to ensure broader access across regions. Health equity remains a global challenge, and Sweden is no exception. The challenge of infrastructure centralization highlights ongoing disparities in access to sequencing services and clinical trials. Although PCM is becoming routine in some healthcare units, positive action is required to improve access and equity. Well-informed clinicians working in peripheral regions are crucial to ensure that all patients benefit from the advances in PCM.

“*Physical access to this doesn’t necessarily mean regional distribution; it’s just about ensuring we have access. Sometimes, thinning out and distributing can be counterproductive.*”

(Senior Researcher, Code 9)

## 4. Discussion

Our study offers insights into how PCM implementation is experienced within a major hub of biomedical research and medical care, providing a rich case-based understanding of the dynamics at play. The key insights that we draw from the analysis are summarized in Table 4.

### 4.1. Summary of Key Findings

The structured interviews revealed that PCM is a complex and evolving concept, with definitions ranging from vague concepts to a transformative cancer care approach. Key findings include the need for leadership, collaboration, and the establishment of adequate organizational, legal, and financial frameworks capable of bridging the gap between academic research and clinical practice. Insights from the analysis clearly indicate that a critical enabler for implementation is shaped by the presence of supportive malmanagement ready to invest in technological infrastructure, break fragmentation, standardized workflows, and introduce new organizational models—such as molecular tumor boards and PCM units. Moreover, long-term political commitment is generally considered vital for ensuring that PCM becomes an inclusive and equitable component of cancer care. The need for long-term, integrated financing is interesting to understand from a global perspective. Literature and insights corroborate this finding, pointing to national programs and comprehensive clinical trials as a solution to mitigate cost-driving factors [24,38,39]. Financial challenges are prevalent; across Europe, the high costs associated with advanced molecular diagnostics and targeted therapies have raised concerns about sustainability within publicly funded healthcare systems [25]. These parallels underscore the necessity for international collaboration to address shared obstacles in PCM implementation while highlighting the importance of tailoring strategies to each country’s specific legal, financial, and organizational contexts.

### 4.2. Organizational Inertia in Different Phases of Translation

The informants placed translational research at the core of PCM implementation. Current models of translational research often refer to a “valley of death,” a gap between early-stage research and clinical application where promising discoveries fail to translate into practice due to structural, financial, and operational barriers [40]. Woolf’s analysis offers a framework for understanding the challenges and opportunities associated with translational research [41]. He identified the T1 (translation from experimental science to humans) and T2 (translation to clinical treatment) phases of the translational process. The current strong emphasis on the T1 phase, which focuses on leveraging basic science to create new clinical interventions, has led to significant advances in the development of novel diagnostics and therapies. Woolf posits that the real challenge often lies in the T2 phase, which ensures that these innovations are implemented effectively in everyday clinical practice. For Woolf, the T2 phase frequently struggles with organizational challenges related to human behavior. Several of our informants provided concrete examples of early clinical trials and implementation efforts while remaining vague on the long-term implications of PCM. This suggests that PCM has made significant strides at the T1 level in this context. However, there are considerable barriers to its widespread adoption in routine healthcare, which falls under the T2 domain. These barriers include “organizational inertia”" (resistance to changes in organizations, structures, strategies, or operations) [42] fueled by a lack of adequate infrastructure and the complexity of integrating data-intensive methods into standard clinical practice. Moreover, our findings underscore the gap between current activities and their desired impact. Following the conception by Drolet et al. of a “biomedical research translation continuum” [43], the focus for many informants remains predominantly on T1 (translation of basic science to humans) and T2 (translation to clinical treatment), while another often-overlooked challenge might rather be in advancing to T3 (translation to practice), which is bringing proven methods to broader scale and levels of implementation. There is consensus among the interviewees that more perspectives on health economics (and analysis) are required, which aligns with Woolf’s [41,44] arguments, broadening the research focus to include T3. Without this shift, progress in translational research remains limited.

### 4.3. Normative Isomorphism and the Emergence of Third-Form Organizations

Our findings highlight a parallel tendency to “normative isomorphism” (the process through which organizations become similar to each other) [45,46] and “organizational inertia” [42] in the context of translational efforts across academia and healthcare institutions. The interviews indicated that, to generate an adequate translational flow, academia must increasingly adopt the healthcare strategy of productivity and standardization. Conversely, healthcare must embrace academia’s dynamism, suggesting that a “third-form organization” is required to bridge the gap between the two institutions. This observation reinforces the insight that while isomorphism enhances mutual understanding between institutions and fosters more effective collaboration, specific differences remain insurmountable. Indeed, academia, the healthcare sector, industry, and patient organizations operate within their primary interests and roles. Although they may use similar terminologies and share objectives, their interpretations, needs, resources, and expectations differ substantially, leading to misalignments that create a fragmented environment despite physical proximity and shared goals, stalling translational progress. A “third-form organization” specifically designed to facilitate translation could mitigate the unavoidable differences while still achieving functional isomorphism.

While isomorphism may foster mutual understanding and alignment, many structural and cultural differences between sectors remain difficult to reconcile. Academia, healthcare, industry, and patient organizations continue to operate according to distinct mandates, incentive systems, and accountability structures. Although they may use overlapping terminology and pursue shared goals, these differences in interpretation, resource allocation, and operational logic often lead to misalignment and hinder integration despite physical proximity and collaborative intent.

A third-form organization could take the form of a joint governance unit, a translational institute, or a purpose-built hybrid structure embedded within both systems. Such an entity would need to possess its own administrative and legal identity while being accountable to both academic and clinical stakeholders. Its feasibility lies in its ability to coordinate across boundaries—managing shared infrastructure (e.g., data platforms or trial networks), standardizing translational workflows, facilitating joint appointments and training programs, and mediating regulatory and ethical compliance. Rather than eliminating differences, it would manage and translate them, enabling functional integration without requiring full institutional convergence. Implementing such a model would require strong leadership commitment, clear mandates, and sustainable resourcing, but it offers a realistic path forward to overcome the recurring bottlenecks in the translation of precision cancer medicine from bench to bedside.

### 4.4. The Findings in an International Perspective

Similar to the challenges identified at KUH and KI, a recent study on the implementation of precision medicine in Europe with diverse public healthcare systems [47] emphasized the importance of stakeholder alignment and coordinated governance, noting that fragmented roles and siloed responsibilities hinder the equitable rollout of precision medicine [28,48]. Another shared challenge across both articles is the misalignment between regulatory frameworks and the fast-paced development of genomic technologies, echoing the European-wide concern that legal complexity hinders the broader adoption of precision medicine initiatives. Another recent qualitative study from a U.S. community oncology setting, a private insurance-based healthcare system, reflected identified barriers to translating genomic advances into routine care due to variability in practitioner readiness and systemic incentives. This reinforces our observation that fostering shared understanding and strategic educational efforts are essential to implementation success [48]. Studies from other contexts corroborate our finding that successful PCM implementation hinges on technological readiness and bridging structural divides between research and care, supported by adaptive governance, long-term political commitment, and patient equity-focused policies.

### 4.5. Supportive Management, Financing, and Outcomes Research

Insights from the analysis clearly indicate that a critical enabler for implementation is shaped by the presence of supportive management ready to invest in technological infrastructure, break fragmentation, standardize workflows, and introduce new organizational models—such as molecular tumor boards and PCM units. Moreover, long-term political commitment is generally considered vital for ensuring that PCM becomes an inclusive and equitable component of cancer care. The need for long-term, integrated financing is crucial and probably of importance across national borders. Literature and insights corroborate this finding, pointing to national programs and comprehensive clinical trials as a solution to mitigate cost driving factors [24,38,39]. Financial challenges are prevalent; across Europe, the high costs associated with advanced molecular diagnostics and targeted therapies have raised concerns about sustainability within publicly funded healthcare systems [25]. These parallels underscore the necessity for international collaboration to address shared obstacles in PCM implementation while also highlighting the importance of tailoring strategies to the specific legal, financial, and organizational contexts of each country.

A critical takeaway from our findings is the importance of outcomes research [49] and organizational analysis in fostering evidence-based organizational self-awareness, which in turn enhances an institution’s capacity for change. In several statements by the informants, the need for health, economic, and organizational evaluations of PCM was stressed. Many of the current obstacles remain unless stakeholders understand their roles and contributions to the translational process. This self-reflection is based on coherent and continuous evaluations of translational research capacity and conditions for implementation, which are essential for overcoming persistent barriers that prevent effective collaboration.

### 4.6. Future Directions

This study identifies the intersection of multiple and diverse kinds of gaps, which could be referred to as a “gap conjuncture” consisting of organizational, legal, and financial obstacles and a lack of clear delineations of responsibilities [50]. These intersecting gaps create challenges in the implementation process, as multiple actors perceive the gaps from different perspectives, leading to confusion about priorities and who is responsible for initiating and driving change [51,52]. This can result in stakeholders feeling “pacified in the gaps,” where the intent to collaborate is evident but practical steps towards collaboration and resilience are unclear or insufficient [53]. Further exploration of the practicalities of collaboration appears to be necessary, particularly to understand how the intention to collaborate can be translated into effective actions. The focus should be on developing collaborative frameworks in which all stakeholders have a shared understanding of PCM and their respective roles in its implementation [54,55].

Future studies should include a broader range of informants to ensure a more comprehensive understanding of the relevant issues. Additionally, a comparative context should be included to assess the contributions of local factors. This approach will help identify common challenges across different environments while also recognizing unique local organizational constraints, ultimately leading to more robust and generalizable findings.

### 4.7. Strengths and Weaknesses of the Study

Inclusion in the research team of both insiders with long-term PCM experience and non-biased co-researchers with no prior contextual experience enhances the validity of this analysis by minimizing potential biases. Two co-authors, defined as insiders, were aware of their potential biases and paid scrupulous attention to the risks by reflecting during the interview analysis and discussing their interpretation with the other co-authors. The use of member checking further enhances credibility by allowing the verification and identification of potential additional biases.

Several limitations may have affected the comprehensiveness of our conclusions. The categories of informants were somewhat restricted, which may have constrained the diversity of perspectives and insights captured. A broader range of professional categories, including decision-makers, biotech representatives, and patient organizations, could have enriched the data, providing a more nuanced understanding of the PCM implementation process. The time gap between the first and last interviews may have influenced the analysis of contextual conditions, particularly in a rapidly evolving field such as PCM. To ensure the validity of the data, 30% of the interviews (*n* = 5) were conducted in June 2024. This confirmed the persistence of the fundamental issues identified in this study.

## 5. Conclusions

PCM implementation requires a complex interplay of technological, organizational, and socio-political factors. This study highlights that while scientific and clinical advances have positioned PCM as a transformative force in oncology, its successful translation into routine clinical practice is hindered by fragmented organizational structures, insufficient collaboration, and the limited integration of research and healthcare services.

Strong leadership, cross-sectoral collaboration, and a supportive policy environment are key enablers. However, persistent challenges, such as regulatory complexity, financial misalignments, and data management constraints, must be addressed through targeted interventions. A “third-form organization” integrating the strengths of academia, healthcare, and industry could mitigate institutional divides and support translational efforts more effectively.

Future efforts should prioritize the development of shared national infrastructure, enabling real-time data sharing, interdisciplinary training, and sustainable funding models. Policymakers must also adopt a long-term perspective, ensuring that legislative frameworks are flexible enough to accommodate technological advancements while safeguarding ethical and equitable patient care. Bridging the gap between research innovation and clinical implementation requires technical solutions and systemic changes driven by collective commitment and a shared vision across stakeholders of patient-centered cancer care.

Insights from this analysis clearly indicate that a critical enabler for implementation is shaped by the presence of supportive malmanagement ready to invest in technological infrastructure, break fragmentation, standardized workflows, and introduce new organizational models—such as molecular tumor boards and PCM units. Moreover, long-term political commitment is generally considered vital for ensuring that PCM becomes an inclusive and equitable component of cancer care. The need for long-term, integrated financing is interesting to understand from a global perspective. The literature and insights corroborate this finding, pointing to national programs and comprehensive clinical trials as a solution to mitigate cost-driving factors [24,38,39]. Financial challenges are prevalent; across Europe, the high costs associated with advanced molecular diagnostics and targeted therapies have raised concerns about sustainability within publicly funded healthcare systems [25]. These parallels underscore the necessity for international collaboration to address shared obstacles in PCM implementation while highlighting the importance of tailoring strategies to each country’s specific legal, financial, and organizational contexts.

The successful implementation of PCM depends on a coherent strategy that addresses both technical and systemic challenges. Our findings highlight the need for a shared national vision to align diverse stakeholder perspectives and foster interdisciplinary collaboration across healthcare, academia, industry, and regulatory bodies. Robust technical infrastructure, including genomic technologies, bioinformatics, and integrated data systems, must be complemented by new organizational models—such as molecular tumor boards and PCM coordination units—that institutionalize interdisciplinary workflows. Modernizing legal and regulatory frameworks is essential to enable data sharing and support translational innovation. Sustainable financing mechanisms that bridge research and clinical care are critical to scale up PCM beyond pilot initiatives. Ensuring patient engagement, ethical oversight, and a commitment to equity at the core of PCM policy will promote inclusive and socially responsible implementation. Together, these policy directions provide a foundation for translating the promise of PCM into clinical reality.

Strategic Recommendations for Advancing Personalized Cancer Medicine

Establish Integrated Governance
  - Create joint leadership structures across academia, healthcare, industry, government, and patient groups.
  - Align priorities, share resources, and ensure inclusive, transparent decision-making.
Create Bridging Organizations
  - Set up hybrid centers or networks linking research and clinical care.
  - Foster collaboration between scientists, clinicians, industry, and patients to accelerate translation.
Invest in shared infrastructure and sufficient funding
  - Build national platforms for data, biobanking, and diagnostics.
  - Ensure equitable access and long-term support through pooled or public-private funding models.
Modernize Policy and Regulation
  - Streamline approvals for genomic tests and therapies.
  - Update legal frameworks to enable secure data sharing and cross-sector collaboration.
Build Collaborative Culture and Capacity
  - Promote interdisciplinary training and staff exchange between research and care.
  - Engage patients as partners in design, governance, and implementation.

## Figures and Tables

**Table 1:** Participating informants by main professional capacity.

Professional Capacity	Number of Informants
Senior Physician ^1^ (translational researcher)	6
Senior Researcher (academic, KI)	4
Academic Leadership (KI)	2
Hospital Management (KUH)	2
Operative management (hospital, KUH)	2
*Total*	16

^1^ Commitments with both KUH and KI.

**Table 2:** Domains explored during the interviews.

The interview guide focused on six key domains:
1. Participants’ understanding of personalized cancer medicine and its implications
2. Experiences with local PCM initiatives and their organizational context
3. Perceived barriers to PCM implementation (e.g., technological, financial, regulatory)
4. Enablers and facilitating conditions (e.g., leadership, infrastructure, collaboration)
5. Ethical and legal considerations, including data governance and equity of access
6. Visions for future development and suggestions for sustainable integration into routine care

**Table 3:** Themes and categories identified by the analysis.

Theme	Categories
Different conceptions of PCM	PCM is associated with methods and technologies, including primarily genomics-based diagnostics using new-generation sequencing (NGS)
	PCM is a form of care that generates and analyzes patients’ molecular biomarkers to guide individualized treatments.
	Precision and personalized medicine are used synonymously with some variation. PCM is not coherently defined.
	PCM is an emerging concept that incorporates new ideas into former principles of cancer medicine.
	The PCM is an established concept with expanding content.
Complex and dynamic relationships between actors	PCM is conditioned by actors that interact with different roles, incentives, and means of development.
	Dynamic cooperation between actors with different motives for change.
	Academic leadership must embody the right competencies, be visionary, and identify suitable organizational structures to achieve the desired change.
Appropriate technologies and structures	Academic leadership must embody the right competencies, be visionary, and identify suitable organizational structures to achieve the desired change.
	Technologies represent great potential, but there are many challenges to their application in practice.
New organizational forms facilitate the implementation	PCM requires new forms of working that entail new types of professionals.
	There is a need for new forms of organization or changes in existing organizations that can mediate cooperation among stakeholders.
	Some structures need to be developed that can influence the implementation of PCM in cooperation between actors.
Political engagement and legislative efforts	The legal conditions are not adapted to current needs, reducing value for patients and society.
	Political interest and engagement determine the conditions for translational research and implementation into healthcare.
Financial preconditions for translational research	PCM drives costs and requires investments by all parties to secure the necessary infrastructures.
	Many funders need more funding, concerted funding, and new funding models.
Patients’ participation, ethics, and equity	Maintaining ethical standards is essential.
	PCM entails increased complexity and an increased challenge for patient participation.
	Equality regarding treatment effects and access to services is yet to be achieved.

**Table 4:** Summary of key findings and insights.

Theme	Finding	Insights
Different conceptions of PCM	Stakeholders hold diverse and sometimes conflicting understandings of PCM	Variability in definitions (“precision” or “personalized” medicine) affects strategic alignment and expectations across clinical, research, and leadership domains. Shared understanding is foundational for coherent implementation.
Complex and dynamic relationships between actors	PCM implementation depends on sound, multi-level, interdisciplinary collaboration	Effective collaboration between healthcare, academia, industry, and regulators is essential. Roles and responsibilities must evolve dynamically. Strong leadership and vision are critical to bridge institutional divides.
Appropriate technologies and structures	Technical capacity and infrastructure are prerequisites for PCM	Advanced technologies (e.g., NGS, bioinformatics) must be embedded within structured workflows. Shared data systems, clinical protocols, and staff training are required for effective operational integration
New organizational forms facilitate the implementation	New organizational models promote the integration of research and care	Molecular tumor boards, cross-institutional PCM units, and novel staff roles (coordinators, informaticians) enable structured interdisciplinary work and institutionalize PCM processes.
Political engagement and legislative efforts	Legislative and policy frameworks critically shape PCM progress	Outdated legal structures and fragmented governance hinder data sharing and innovation. Political will, regulatory updates, institutional support, and national strategies are needed for translational initiatives to progress.
Financial preconditions for translational research	Sustained funding models are necessary for scalability	PCM requires high-cost investments that exceed traditional budgets. Gaps between research funding and clinical operations need bridging. Long-term, integrated financing and value-based assessments are vital.
Patients’ participation, ethics, and equity	Patient engagement and equity are central ethical imperatives	Patient engagement, patient-reported outcomes, and access to molecular diagnostics and treatments must be addressed. Regional disparities highlight the need for national coordination and inclusion. Ethical governance and advocacy structures support equitable implementation.

## Data Availability

Anonymized raw data supporting the conclusions of this article will be made available by the authors upon request.

## Supplementary Materials

The following supporting information can be downloaded at: https://www.mdpi.com/article/10.3390/jpm15040150/s1. File S1: COREQ Guidelines; File S2: Interview Guide; File S3: Themes, Categories and Sub-Categories.

## Abbreviations

The following abbreviations are used in this manuscript:

PCM	personalized cancer medicine
PM	precision medicine
KUH	Karolinska University Hospital
KI	Karolinska Institutet
RS	Region Stockholm
