# Supplementary material for: Implementing Personalized Cancer Medicine: Insights from a Qualitative Interview Study
Source: J Pers Med. 2025 Apr 9;15(4):150. doi: 10.3390/jpm15040150 (PMC12029028; doi:10.3390/jpm15040150)
Supplement: Supplementary file 1 [file jpm-15-00150-s001.zip › File S1. COREQ Guidelines - Masucci et al.pdf]

File S1. Consolidated criteria for reporting qualitative studies (COREQ) Checklist[1]

Masucci et al. Implementing Personalized Cancer Medicine: Insights from a Qualitative Interview Study. 2025

Domain 1: Research team and reflexivity

Personal Characteristics

1. **Interviewer:** The first author (Michele Masucci) conducted all interviews. His credentials include expertise in PCM implementation and training in qualitative research.

2. **Credentials:** Within the study team in collaboration with the first author (Michele Masucci) holds academic qualifications in medical management and oncology.

3. **Occupation:** Michele Masucci is a PhD student at Karolinska Institutet and a professional in PCM implementation research through the PCM Program.

4. **Gender:** The interviewer is male however it is not deemed relevant for the study.

5. **Experience and training:** Michele Masucci has significant experience in PCM research and guidance in qualitative methodologies, ensuring sufficient expertise in conducting interviews.

Relationship with Participants

6. **Relationship established:** In some cases the interviewer and participants were prior work aquantiances. However no direct employment nor dependencies.

7. **Participant knowledge of the interviewer:** Participants were informed about the interviewer's role in the study, research background, and purpose of the study.

8. **Interviewer characteristics:** The interviewer's professional role and research expertise in PCM were disclosed, which may have influenced participant responses. Mainly assumptions due to familiarity of topic beteween interviewer and informant nmight have influenced the character of the interview.

---

[1] Allison Tong, Peter Sainsbury, Jonathan Craig. Consolidated criteria for reporting qualitative research (COREQ): a 32-item checklist for interviews and focus groups. International Journal for Quality in Health Care, Volume 19, Issue 6, December 2007, Pages 349 357, https://doi.org/10.1093/intqhc/mzm042

Domain 2: Study Design

Theoretical Framework

9. **Methodological orientation and theory:** The study utilized an inductive approach and conventional content analysis for qualitative data analysis.

Participant Selection

10. **Sampling:** Purposeful sampling was used to select participants with diverse roles in PCM implementation.

11. **Method of approach:** Participants were contacted via email based on their involvement in PCM-related implementation activities.

12. **Sample size:** Twenty participants were contacted, and sixteen consented to participate.

13. **Non-participation:** Four participants declined or were unable to participate, due to lack of response to the invitation. No reasons where available to be disclosed in the report.

Setting

14. **Setting of data collection:** Interviews were conducted either in person or via online videoconferencing, depending on participant preference.

15. **Presence of non-participants:** Only the interviewer and participant were present during interviews.

16. **Description of sample:** Participants included senior physicians, researchers, middle and senior managers at Karolinska Institutet and Karolinska University Hospital.

Data Collection

17. **Interview guide:** A semi-structured interview guide was used, with questions addressing factors influencing PCM implementation. The interview guide was pilot tested.

18. **Repeat interviews:** Repeat interviews were not conducted.

19. **Audio/visual recording:** All interviews were digitally recorded for audio.

20. **Field notes:** The use of field notes was carried out but not explicitly mentioned in the manuscript.

21. **Duration:** Interviews lasted 40–60 minutes.

22. **Data saturation:** Data collection ceased once thematic saturation was achieved.

23. **Transcripts returned to participants:** Themes and the draft manuscript were shared with participants for member checking, allowing them to review and endorse the findings. All informants endorsed the findings.

Domain 3: Analysis and Findings

Data Analysis

24. **Number of data coders:** Three authors independently coded the data and collaboratively analyzed themes.

25. **Description of the coding tree:** Codes were derived inductively and categorized into themes through consensus among the research team.

26. **Derivation of themes:** Themes were derived from the data using an inductive approach to content analysis.

27. **Software:** Microsoft Excel and MIRO® were used to organize and analyze data. Data was stored in OneDrive Microsoft Environment at Karolinska Insitututet.

28. **Participant checking:** Participants reviewed the draft analysis, themes, and manuscript to validate findings and provide additional feedback.

Reporting

29. **Quotations presented:** Representative quotations are provided for each theme to support the findings.

30. **Data and findings consistent:** Themes and findings are consistent with the data presented.

31. **Clarity of major themes:** Major themes are clearly identified and described in the results section.

32. **Clarity of minor themes:** Minor themes are incorporated within broader categories to provide a comprehensive analysis.
