# Supplementary material for: Implementing Personalized Cancer Medicine: Insights from a Qualitative Interview Study
Source: J Pers Med. 2025 Apr 9;15(4):150. doi: 10.3390/jpm15040150 (PMC12029028; doi:10.3390/jpm15040150)
Supplement: Supplementary file 1 [file jpm-15-00150-s001.zip › File S2. Interview Guide - Masucci et al.pdf]

Questionnaire: Conditions for Translational Cancer Research
Informants: oncologists, cancer nurses, caregivers, cancer researchers, decision-makers in cancer research and/or healthcare, representatives from pharmaceutical companies and the biotech industry, patient representatives.

BACKGROUND
I am conducting a study on the conditions for translational cancer research with a special focus on the concept of precision medicine or personalized medicine in cancer. The purpose is to examine how cancer research and cancer care are conducted, specifically regarding the introduction of precision medicine and any changes it has brought in translating discoveries to clinical practice. You have been asked to participate due to your role in this area of cancer research and healthcare. Questions will relate to your experiences and thoughts on cancer research and healthcare. All participants in the study who are interviewed will remain anonymous, and I will anonymize all interview responses. You have the right to request access to or deletion of your data and responses saved for the purpose of this study at any time. You also have the right to access the transcription of this interview and be informed about how responses are analyzed and used in the study. If requested, a final manuscript can be sent to you before publication. Anonymization will be applied to direct quotes, and you will be consulted beforehand.

INTRODUCTION

- Do you have any questions about the study and the interview that we are about to start?
- What is your relationship to cancer research or healthcare?
- What are your job responsibilities and duties?
- How long have you been in this role?

HISTORY/CONCEPT

- How would you define:
    - Precision medicine in cancer
    - Personalized medicine in cancer
    - Individualized treatment in cancer
    - Targeted therapy in cancer
    - Molecular diagnostics in cancer
    - Translational research

- How does precision medicine in cancer differ from established treatment concepts?
- Can we expect other concepts to replace precision medicine?
- What related concepts exist and how do they differ from precision medicine?

SCIENCE
- What discoveries underlie precision medicine?
- What developments do you expect in the near future?

TECHNOLOGY

- What kinds of technologies underlie precision medicine in cancer?
- How do these technologies impact research/healthcare?

ACTORS

- Who are the primary actors within PCM?
- Which decision-making authorities and organizations affect PCM?
- How do they relate to each other?
- What influence do they have?

ORGANIZATION

- How should the translational process be organized, in your opinion?
- What type of organization is best suited for PCM?

- What kind of leadership is required for the introduction of PCM?
- Is special leadership required for the introduction of PCM?

- What are the primary barriers you currently see?
- What are the primary conditions that exist today?

- What changes are needed to improve the current situation?
- What decisions have been made recently that affect PCM?
- What legislation is needed/lacking that affects PCM?

FUNDING

- How does precision medicine in cancer affect established funding models for research and healthcare?
- Are there special funding requirements for precision medicine in cancer?
- Which actors finance precision medicine in cancer today?
- Are new actors needed for financing precision medicine in cancer?

ETHICS

- Are there special ethical considerations for precision medicine?
- Who has access to precision medicine today?
- Does precision medicine affect health inequalities within cancer care? If yes, how?
- Are there groups that particularly benefit from the introduction of precision medicine?

PATIENT PARTICIPATION

- Does the patient's influence on their own treatment with precision medicine in cancer change?
- If yes, how?

ANY OTHER QUESTION YOU THINK HAS BEEN MISSED?
