# Supplementary material for: Implementing Personalized Cancer Medicine: Insights from a Qualitative Interview Study
Source: J Pers Med. 2025 Apr 9;15(4):150. doi: 10.3390/jpm15040150 (PMC12029028; doi:10.3390/jpm15040150)
Supplement: Supplementary file 1 [file jpm-15-00150-s001.zip › File S3. Themes, Categories and Sub-Categories.pdf]

File S3. Themes, categories, and sub-categories identified by the analysis.

Theme	Category	Sub-Category
**Different conceptions of PCM**	PCM is strongly associated with specific methods and technologies, which include primarily genomics-based diagnostics using new-generation sequencing (NGS).	PCM is primarily associated with genomics-based diagnostics.
		PCM is biomolecular characterization (profiling).
		PCM is primarily associated with access to data generated by next-generation sequencing technologies.
		Specific discoveries and treatments have been paradigmatic for PCM.
		PCM has a prehistory and has emerged gradually.
	PCM is a form of care that generates and analyzes patients' molecular biomarkers to guide individualized treatments.	Molecular genomic characterization and mutation analysis targets are primary associations
		Traditional definitions are emphasized, such as "the right treatment at the right time" and tailoring the treatment to the individual.
		PCM implies treatment centered around the specific needs and preferences of the patient or individual.
		PCM entails an improvement in diagnostics and treatment.
		PCM is more data intensive.
	Precision and personalized medicine are used synonymously with some variation.	PCM is associated with the development of genomics as a diagnostic method.
		Personalized and precision medicine are synonymous definitions.
		Personalized medicine includes more aspects than precision medicine.
		Precision cancer medicine is the appropriate concept.
		Personalized cancer medicine is the appropriate concept.
	PCM is an emerging concept that incorporates new ideas into former principles of cancer medicine.	That which PCM entails existed before the concept emerged.
		There are other concepts related to PCM.
	PCM is not coherently defined.	PCM is a vague concept that has become popular.
		There is not much substance to the concept.
		There are things associated with PCM which are not PCM.
	The PCM is an established concept with expanding content.	There are established definitions by authorities and public health organizations.
		PCM includes more than what it usually is associated with.
**Complex and dynamic relationships between actors**	PCM is conditioned by actors that interact with different roles, incentives, and means of development.	Multiple actors have been identified as playing pivotal roles in implementing PCM.
		Engagement from the pharmaceutical and biotech industry is essential while maintaining in-house capability. There are significant differences in the collaboration mode between small, medium, and large corporations.
		The main stakeholders are the patients.
		Collaboration across key parties is necessary, and there are conditions for it; however, there are also fundamental challenges.
	Dynamic cooperation between actors with different motives for change.	Specific measures that facilitate implementation of PCM.
		Attitudes, culture, and perspectives influence the drive for change.
		The main actors can be described in a Penta helix model with concerted action between the following main actors: industry, academy, healthcare, government, and civil society (patient organizations).
	Academic leadership must embody the right competencies, be visionary, and identify suitable	Change requires leadership; there is a lack of clear mandate and direction. New strategies are underway. However, change takes time, and agreements need to come into effect.

	organizational structures to achieve the desired change.	There is a need for new forms of visionary leadership. Self-awareness of the organization leads to being able to create new conditions.
		Leadership needs engagement and knowledge that gathers actors to collaborate.
**Appropriate technologies, structures, and organizations**	Organizational arrangements are needed to interface actors in pursuit of specific results. Clinical trial capabilities should expand, new roles and competencies are needed	There is a need for competencies and advancements across stakeholders. Education and capacity for education are missing. Cooperation across stakeholders on IT resources needs to be in place.
		New roles and categories of professionals need to be recruited.
		Conditions for the further development and implementation of PCM.
		Clinical trial organizations are important in the implementation of PCM. There is a division between routine healthcare and translational research.
	Technologies represent great potential, but there are many challenges to their application in practice.	There is a need to coordinate methodologically and conceptually the development of shared infrastructures for data management and computing, as well as share/pool resources across stakeholders.
		Necessary competencies and infrastructures for research are available; however, specific capabilities, expertise, and functions should be developed.
		Emerging technologies will reshape the landscape of PCM. In many instances, rapid development is occurring.
**New organizational forms**	PCM requires new forms of working that entail new types of professionals.	There is a need for organizations that foster collaboration to aid in introducing PCM into healthcare.
		PCM requires further education and new ways of working, such as multidisciplinary cooperation.
	There is a need for new forms of organization or changes in existing organizations that can mediate cooperation among stakeholders.	Organizational culture needs to foster scientific vision building and support bottom-up initiatives. This needs to be paired with research data infrastructures and organizations such as CCCs that can deliver specific services such as legal counsel familiar with the scientific conditions.
		There is a need for a public authority to function as a national organ for translation and implementation, which will modernize healthcare.
	There are structures in place and structures that need to be developed that can influence the implementation of PCM in cooperation between actors.	The conditions within healthcare are not optimally designed for translational research. Academic research is a driving force with more room to maneuver but does not have the mandate to perform health care.
		The role of academic institutions is to develop and for industry to transform new findings into products. Private healthcare actors are not too engaged in research.
		Sweden is too small for regional competition, and national coordination would be beneficial.
		There are challenges to structural change. Fragmentation is a problem. PCM is difficult to study and implement in smaller regions. Active measures are necessary to counteract the uneven distribution of resources for PCM.
**Political engagement and legislative efforts**	The legal conditions are not adapted to current needs, reducing value for patients and society. Legal reforms can facilitate translational research.	Legislation for biobanking needs reforms to facilitate PCM.
		Patient integrity and security are fundamental. Legislation needs to be developed. The interpretation of rules is difficult, which can produce a conservative stance blocking progress. Support for researchers is necessary to find solutions that adhere to laws and regulations without becoming organizationally unfeasible.

		Sharing data is important for research and PCM. Securing patient integrity is essential. Infrastructures and legal reforms are required to facilitate data sharing for research purposes while maintaining security and patient integrity. Collecting large datasets poses ethical challenges. Data security is important to maintain.
		There are legal challenges because public entities cannot accumulate profits to fund research and development. Opportunities are lost in the gap.
		There needs to be an infrastructure with legal experts that can proactively manage and prepare for requests for secondary use of clinical data for research purposes. Wet lab infrastructures need legal support to adhere to accreditation.
	Political interest and engagement determine the conditions for translational research and implementation into healthcare.	More engagement and political will are needed to advance the conditions for healthcare and research. Implementing PCM requires political support and long-term understanding to achieve the necessary changes. Regional policy and centralization are recurring issues. Management needs to have a better understanding of the needs of medical experts. Politicians are ultimately responsible for creating the necessary preconditions.
		Benchmarking with other institutions is needed to understand translational research performance. The Swedish model and organizational culture for research and health care have unique characteristics.
		There are international role models. International collaboration develops local PCM capabilities. Sweden is a small country that depends on international involvement for progress.
**Financial preconditions for translational research**	PCM drives costs and requires investments by all parties to secure the necessary infrastructures.	PCM comprises high costs and implies a challenge in how the different funders allocate resources to realize PCM capacity.
		Academic research suffers from structural hindering factors that negatively shape the conditions for funding.
		Healthcare systems fail to integrate academic research. There is a need for an academically driven infrastructure that can transcend institutional objectives and forms of funding.
	Many funders need more funding, concerted funding, and new funding models.	Academic project funding is a primary contributor to the current translation of PCM into healthcare. Research funders also need to think long-term and promote implementation efforts within health care.
		There are different funders, from public to private, and there are industry sponsorships. Public research funds primarily support research. Private research funds are oriented toward basic science and not for clinical trials.
		Health economic studies on PCM are needed to identify returns of investments and secure cost efficiency.
**Patients' participation, ethics, and equity**	Maintaining ethical standards is essential.	There are ethical questions that need to be addressed on a national level.
		Incidental findings are a substantial question: How can family members be informed, and should patients learn about anomalies? Using a patient's biological data is sensitive, and anonymization of genetic information is difficult.
		Ethical permits and patient consent are necessary for clinical trials.
	Patient consent and participation are essential for a data-intensive form of care. PCM entails increased complexity and an increased challenge for patient participation.	Patients' participation is central for PCM and research in general. Patients have become more active, which requires educational efforts and information directed to the public.
		The increased use of patient data with PCM requires patient consent for secondary use for research purposes. Patient consent is essential. Universal consent forms facilitate research.
	Equality regarding treatment effects and access to services is yet to be achieved.	Centralization of infrastructures is a precondition for universal access. Organizational solutions will provide PCM with health equality and equity.
		Clinical trials are a precondition to access PCM today.

		PCM is a motor for equality.
		PCM principles and methods are already part of routine healthcare; however, information and research are necessary to increase access, equality, and democratization.
		Access to more information does not always equate with better treatment outcomes. PCM, in general, provides better outcomes for smaller patient groups that are medically motivated.
